# Neural Correlates of Threat Perception: Neural Equivalence of Conspecific and Heterospecific Mobbing Calls Is Learned

**DOI:** 10.1371/journal.pone.0023844

**Published:** 2011-08-29

**Authors:** Marc T. Avey, Marisa Hoeschele, Michele K. Moscicki, Laurie L. Bloomfield, Christopher B. Sturdy

**Affiliations:** 1 Department of Psychology, University of Alberta, Edmonton, Alberta, Canada; 2 Department of Psychology, Algoma University, Sault Ste. Marie, Ontario, Canada; 3 Department of Psychology, Centre for Neuroscience, University of Alberta, Edmonton, Alberta, Canada; Claremont Colleges, United States of America

## Abstract

Songbird auditory areas (i.e., CMM and NCM) are preferentially activated to playback of conspecific vocalizations relative to heterospecific and arbitrary noise [1]–[2]. Here, we asked if the neural response to auditory stimulation is not simply preferential for conspecific vocalizations but also for the information conveyed by the vocalization. Black-capped chickadees use their *chick-a-dee* mobbing call to recruit conspecifics and other avian species to mob perched predators [3]. Mobbing calls produced in response to smaller, higher-threat predators contain more “D” notes compared to those produced in response to larger, lower-threat predators and thus convey the degree of threat of predators [4]. We specifically asked whether the neural response varies with the degree of threat conveyed by the mobbing calls of chickadees and whether the neural response is the same for actual predator calls that correspond to the degree of threat of the chickadee mobbing calls. Our results demonstrate that, as degree of threat increases in conspecific chickadee mobbing calls, there is a corresponding increase in immediate early gene (IEG) expression in telencephalic auditory areas. We also demonstrate that as the degree of threat increases for the heterospecific predator, there is a corresponding increase in IEG expression in the auditory areas. Furthermore, there was no significant difference in the amount IEG expression between conspecific mobbing calls or heterospecific predator calls that were the same degree of threat. In a second experiment, using hand-reared chickadees without predator experience, we found more IEG expression in response to mobbing calls than corresponding predator calls, indicating that degree of threat is learned. Our results demonstrate that degree of threat corresponds to neural activity in the auditory areas and that threat can be conveyed by different species signals and that these signals must be learned.

## Introduction

Bird calls, unlike songs, are a relatively understudied communication system in behavioural neurobiology [5]. Calls serve numerous functions including signaling potential threats which are a primary concern for many species. Threat signals often involve complex behaviour that requires learning both the nature and degree of the potential threat [6]. Such complex acoustic signaling systems are used to convey information about potential threats to conspecifics or heterospecifics [3], [4], [6]–[8]. Black-capped chickadees use a sophisticated vocal signaling system to indicate the type and degree of potential threat [4]. Black-capped chickadees use a high frequency, low amplitude *high zee* call to indicate the presence of an aerial predator, and a loud, complex *chick-a-dee* mobbing call to recruit conspecifics and other avian species to mob a perched predator [3], [4], [9]–[11]. Templeton et al. (2005) demonstrated that the structure of the black-capped chickadee *chick-a-dee* mobbing call encodes the degree of threat of potential predators [4]. Generally, mobbing calls produced in response to smaller, higher-threat predators contain more “D” notes compared to those produced in response to larger, lower-threat predators. However, where and how the degree of threat is encoded in the brain is unknown.

Auditory processing nuclei in songbirds, such as the caudomedial mesopallium (CMM) and caudomedial nidopallium (NCM), putatively perform functions similar to those of the mammalian auditory cortex [2], [12], [13]. These regions may activate in response to degree of threat because they activate in response to complex auditory information [1], [2], [12]–[14]. Use of the immediate early gene ZENK (zif-268, egr-1, NGFI-A, or Krox-24) as a regional activity marker has established CMM and NCM as crucial in processing complex auditory information such as conspecific vocalizations [1]. In general, conspecific vocalizations induce more ZENK positive cells in CMM and NCM compared to heterospecific vocalizations and tones that induce fewer ZENK positive cells [13]. However, the conspecific signals used as playback stimuli are, necessarily, songs that are biologically relevant to the species' natural history. In contrast, heterospecific signals are often songs of other species that are not biologically relevant signals to the species being studied. In some situations, however, heterospecific vocalizations may be more salient than conspecific vocalizations and this may be reflected in the corresponding neural activity.

**Figure 1:**
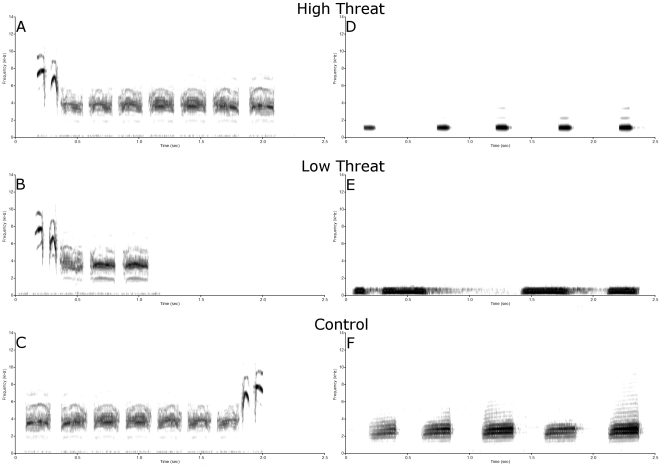
Example calls from the 6 playback conditions: y-axis = frequency (kHz); x-axis = time (sec). (A) A black-capped chickadee mobbing call produced in response to a northern saw-whet owl mount (high threat). (B) A black-capped chickadee mobbing call produced in response to a great-horned owl mount (low threat). (C) The reversed black-capped mobbing call from *(A)* (methodological control). (D) A northern saw-whet owl call (high threat). (E) A great-horned owl call (low threat). (F) A red-breasted nuthatch call (threat control).

We investigated whether the degree of threat perceived by black-capped and mountain chickadees is correlated with ZENK activity in CMM and NCM. To achieve this, we played back one of six stimulus types: four threat stimuli and two control stimuli (Fig. 1 a–f), to either wild-caught adult black-capped or mountain chickadees. Thus, we extended the concept of degree of threat not only to differences in the mobbing calls of black-capped chickadees heard by a conspecific [4], and mobbing calls of black-capped chickadees heard by a heterospecific (mountain chickadees) [9], but also to the heterospecific calls of predators that induced these mobbing calls. We used two degrees of threat: high threat (Fig. 1 a, d) and low threat (Fig. 1 b, e). Each degree of threat was conveyed by two distinct signals that shared the same referent, either chickadee mobbing calls to a predator or the corresponding predator calls. The calls of a red-breasted nuthatch (Fig. 1. f), a heterospecific that flocks with both black-capped and mountain chickadees, was used as a control for threat. Reversed mobbing calls (Fig. 1 c) were used as a control to match for spectral and temporal complexity in the *chick-a-dee* mobbing call. To our knowledge, whether two signals from different classes of producers can both convey such complex information as degree of threat, and whether these two signals would produce similar amounts of ZENK expression in the brain, have not been tested. This design allowed us to determine whether the degree of threat is encoded in a neural response in CMM and NCM and whether the ZENK expression levels differ depending on the species specificity of the call (conspecific versus heterospecific).

## Results

### Wild-adult Chickadees

**Figure 2:**
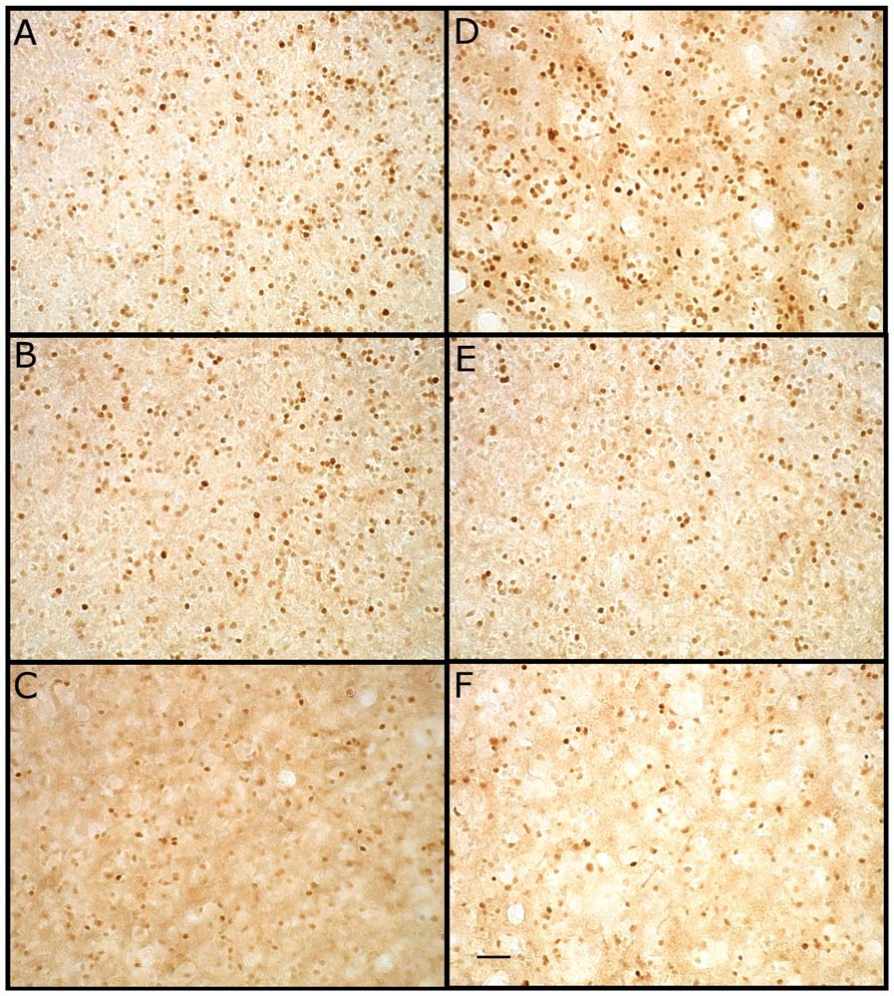
Example ZENK expression in the caudomedial mesopallium of black-capped chickadees to each of the six playback conditions. (A) Black-capped chickadee mobbing calls made to a northern saw-whet owl mount (high threat). (B) Black-capped chickadee mobbing calls made to a great-horned owl mount (low threat). (C) Reversed black-capped mobbing calls from *(A)* (methodological control). (D) Northern saw-whet owl calls (high threat). (E) Great-horned owl calls (low threat). (F) Red-breasted nuthatch calls (threat control). Scale bar 25 µm.

We quantified the number of ZENK positive cells in CMM and the dorsal (NCMd) and ventral (NCMv) portions of NCM in both hemispheres (Fig. 2). We conducted a repeated measures analysis of variance (RMANOVA) with Brain Area×Hemisphere as within subject factors and Listener Species×Playback Condition (Fig. 1) as between subject factors. The amount of ZENK expression varied significantly among Brain Areas [RMANOVA: *F*_2, 48_ = 7.59, *P*<0.01; CMM, M = 103.23, SD = 3.14; NCMd, M = 105.68, SD = 2.89; NCMv, M = 92.5, SD = 2.62]. Pairwise comparisons (Bonferroni corrected) revealed that CMM and NCMd both had significantly more ZENK expression than NCMv (*P* = 0.03; *P*<0.01). There was no significant interaction between Brain Areas and Playback Condition [RMANOVA: *F*_10, 48_ = 1.27, *P* = 0.27]. There was no significant difference between Hemispheres [RMANOVA: *F*_1, 24_ = 0.54, *P* = 0.47], and there was no significant interaction between Hemisphere and Playback Condition [RMANOVA: *F*_5, 24_ = 0.43, *P* = 0.82].

There was no significant difference in the amount of ZENK expression between black-capped and mountain chickadee Listener Species [RMANOVA: *F*_1, 24_ = 0.72, *P* = 0.40], indicating that conspecific and heterospecific mobbing calls induced similar ZENK expression in these closely related species. ZENK expression differed significantly among Playback Conditions for both black-capped and mountain chickadees [RMANOVA: *F*_5, 24_ = 89.57, *P*<0.01], and there was no significant interaction between Listener Species and Playback Condition [RMANOVA: F_5,24_ = 0.85, *P* = 0.53]. Below we analyze the differences between playback conditions by pooling the black-capped and mountain chickadees groups.

**Figure 3:**
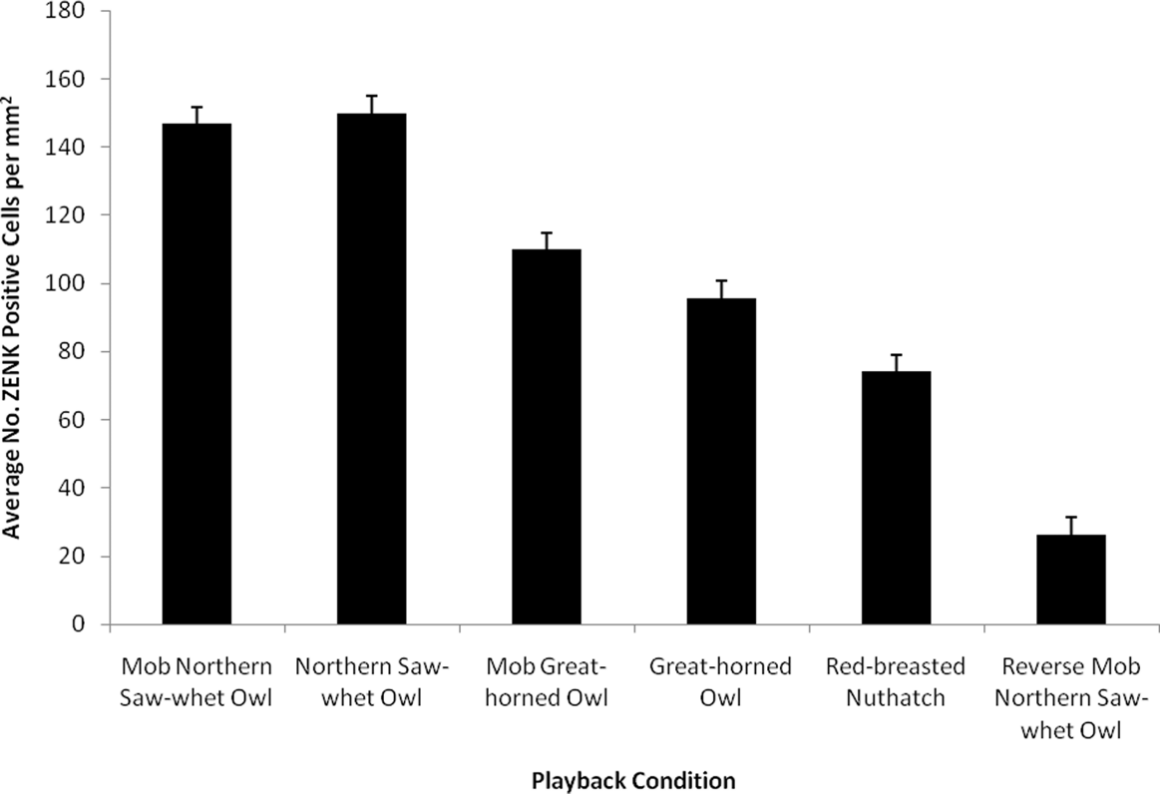
ZENK expression increased in response to higher threat signals and did not vary based on the signal producer. ZENK expression significantly differed among all playback conditions except: mobbing calls to a northern saw-whet owl and northern saw-whet owls calls (*P* = 0.99), and mobbing calls to a great-horned owl and great horned owl calls (*P* = 0.35).

Post-hoc comparisons (Tukey HSD) for Playback Condition indicated that playback of black-capped chickadee mobbing calls produced in response to the high threat northern saw-whet owl generated significantly more ZENK expression than black-capped chickadee mobbing calls produced in response to the low threat great-horned owl (*P*<0.01; Fig. 3). Thus, mobbing calls associated with higher threat generated more ZENK expression than mobbing calls associated with lower threat. Similarly, playback of the high threat northern saw-whet owl calls generated significantly more ZENK expression than the low threat great-horned owl calls (*P*<0.01; Fig. 3). Thus, the degree of threat, whether signaled by chickadee mobbing calls or predator calls, results in higher levels of ZENK expression for high threat signals, independent of whether the signal is produced by a chickadee or predator.

Post-hoc comparisons (Tukey HSD) for Playback Condition indicated that playback of black-capped chickadee mobbing calls produced in response to the high threat northern saw-whet owl did not differ significantly in ZENK expression from playback of northern saw-whet owl calls (*P* = 0.99; Fig. 3). Similarly, ZENK expression following playback of black-capped chickadee mobbing calls produced in response to the low threat great horned owl calls did not differ significantly from ZENK expression following playback of great-horned owl calls (*P* = 0.35). Although each threat level had two distinct signals, one a chickadee mobbing call and one an owl call, there was no significant difference in the amount of ZENK expression induced within a threat level. This result suggests that degree of threat is driving the ZENK expression in CMM and NCM, and not species-specificity. All of the high and low threat playback conditions (mobbing calls and owl calls) differed significantly from both control conditions. The threat control, red-breasted nuthatch calls, generated significantly more expression than the methodological control, reversed chickadee mobbing call (*P*<0.01; Fig. 3).

### Hand-reared Chickadees

ZENK expression levels in black-capped chickadee auditory perception nuclei vary between high and low threat signals but not between different types of signals conveying the same degree of threat. Our next step was to determine whether experience was necessary for the perception of degree of threat in these brain nuclei. To address this question, we hand-reared black-capped chickadees in colony rooms alongside adult chickadees. Hand-reared birds had no experience with either owl species or red-breasted nuthatches. We played back stimuli from one of four conditions to adult hand-reared black-capped chickadees: black-capped chickadee mobbing calls produced in response to a northern saw-whet owl (high threat conspecific), reversed black-capped chickadee mobbing calls to a northern saw-whet owl (control), northern saw-whet owl calls (high threat heterospecific), and red-breasted nuthatch calls (control; Fig. 1 a, c, d, f). Comparing ZENK expression following playback of black-capped chickadee mobbing calls produced in response to a northern saw-whet owl with the ZENK expression following playback of northern saw-whet owl calls allowed us to determine whether experience with predators modulates the number of ZENK positive cells in CMM and NCM.

We conducted a RMANOVA with Brain Area×Hemisphere as within subject factors and Playback Condition as the between subjects factor. In common with the results from wild-caught adult chickadees, results for hand-reared chickadees indicated that the amount of ZENK expression varied significantly among the brain areas. The RMANOVA revealed a significant main effect for Brain Area [*F*_2, 24_ = 9.94, *P*<0.01; CMM, M = 69.55, SD = 3.44; NCMd, M = 66.96, SD = 4.92; NCMv, M = 54.33, SD = 2.82], with more ZENK expression in CMM and NCMd. There was no significant interaction between Brain Areas and Playback Condition [RMANOVA: *F*_6, 24_ = 0.23, *P* = 0.96]. There was no significant difference between Hemispheres [RMANOVA: *F*_7, 84_ = 0.19, *P* = 0.48], and there was no significant interaction between Hemisphere and Playback Condition [RMANOVA: *F*_3,12_ = 0.52, *P* = 0.68].

**Figure 4:**
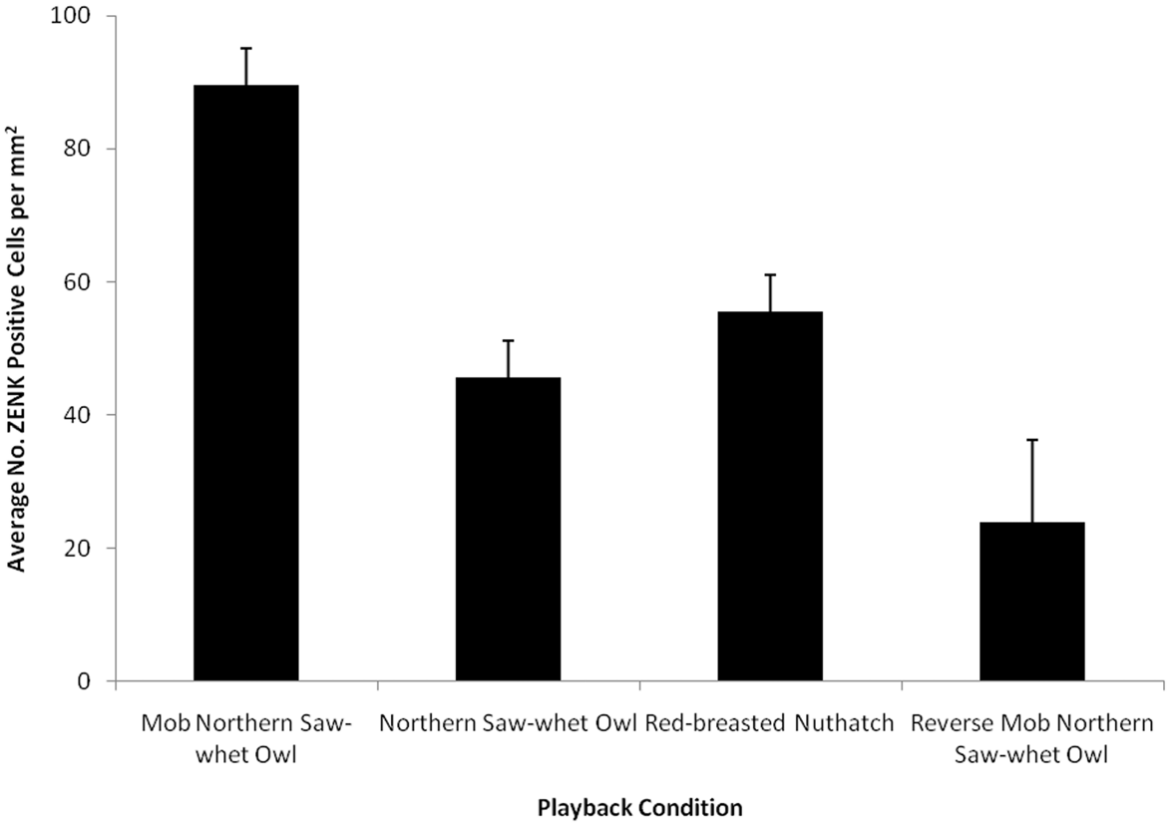
ZENK expression increased in response to conspecific calls but not threat in hand-reared black-capped chickadees. There was no significant difference in the amount of ZENK expression in response to northern saw-whet owl calls (high threat) and red-breasted nuthatch calls (threat control; *P* = 0.44).

The amount of ZENK expression also varied significantly between Playback Conditions [RMANOVA: *F*_3, 12_ = 14.80, *P*<0.01]. Post-hoc comparisons (Tukey HSD) indicated that playback of black-capped chickadee mobbing calls produced in response to the high threat northern saw-whet owl generated significantly more ZENK expression than playback of either northern saw-whet owl calls or red-breasted nuthatch calls (both *P*<0.01; Fig. 4). ZENK expression elicited by playback of northern saw-whet owl calls did not differ significantly from that elicited by playback of red-breasted nuthatch calls (*P* = 0.44; Fig. 4). Unlike in wild-caught adult chickadees, ZENK expression levels in CMM and NCM in hand-reared black-capped chickadees, differ between the two high-threat signals (mobbing calls and predator calls), suggesting that perception of threat level is learned.

## Discussion

In summary, we found that an increased number of ZENK positive cells correspond to increased degree of threat regardless of the producer species or the spectral and temporal features of the signal. In contrast to wild-caught adult chickadees, hand-reared chickadees responded to conspecific mobbing calls with an increased number of ZENK positive cells, but the number of ZENK positive cells did not vary between heterospecific predator and heterospecific non-predator calls. The activation patterns of ZENK positive cells in wild-caught adults and hand-reared black-capped chickadees support the idea that degree of threat is learned and that learning creates changes in the neural activation within CMM and NCM.

The black-capped chickadee mobbing call is a multi-note, broad band vocalization with complex harmonics [3], [4], [15], which is a striking contrast to the simple, tonal vocalization of the northern saw-whet owl call [16]. Although the structure and duration of the signals differ, the mobbing calls and the corresponding owl calls still generated the same amount of activation in CMM and NCM of black-capped chickadees. Despite that the owl calls are not used to signal threat to heterospecifics the wild-caught adult chickadees intercepting the owl calls perceive them as conveying the same degree of threat as the corresponding mobbing calls. The similar patterns of activation between chickadee and owl calls strongly support the idea that CMM and NCM are responding not only the producer or the spectral and temporal properties of the signal but also to the degree of threat associated with the signal.

Previous research initially reported differences in the amount of ZENK expression in CMM and NCM in response to conspecific and heterospecific vocalizations [1]. Subsequently, researchers have used heterospecific signals as a control in ZENK expression studies [17], but here we show that heterospecific signals can generate as much, if not more, ZENK expression depending on the information contained within, or the relevance of, that signal. We build upon previous studies by demonstrating that CMM and NCM do not simply respond in a graded fashion to conspecific and heterospecific signals, but that biological relevance of the stimuli can supersede the conspecific versus heterospecific signals distinction. We confirmed this idea with two closely-related species of chickadee, one that heard conspecific vocalizations and one that heard heterospecific vocalizations of mobbing calls as well as both species hearing heterospecific predator calls.

Chickadees have a sophisticated alarm call system for signaling threat. This study demonstrates that the information conveyed in the signal, the degree of threat, produces a differential response in the auditory perception nuclei we investigated. By studying this system, we were able to show that a conceptual category, such as threat, can be conveyed with very distinct stimulus types that differ in the species of the producer and the signal structure itself. In addition, by studying both wild and hand-reared chickadees, we showed that the degree of threat predators pose is learned, and this learning can be detected in the neural activity patterns of the auditory nuclei.

## Materials and Methods

### Subjects

For this experiment we used 18 wild caught black-capped chickadees (12 male, 6 female), 18 wild caught mountain chickadees (12 male, 6 female), and 16 adult hand-reared black-capped chickadees (7 male, 9 female). Adult black-capped and mountain chickadees were captured from several regions within Edmonton, Alberta, Canada (53°32′N, 113°29′W) and Kananaskis Country, Alberta, Canada (51°02′N, 115°03′W). Hand-reared black-capped chickadees were collected from four different broods (approximately 5–14 days post-hatch) within several regions of Edmonton, Alberta, Canada in June 2004 and June 2005 [18]. Adult black-capped and mountain chickadees were housed individually in cages in conspecific colony rooms immediately after being brought into the lab. Hand-reared black-capped chickadees were transferred into individual cages in either black-capped our mountain chickadee rearing colony rooms at approximately 35 days of age. Food and water was provided *ad libitum* and colony room temperatures were maintained at about 20°C with the natural seasonal light cycle for Edmonton. All studies were conducted in accordance with the Canadian Council on Animal Care Guidelines and policies with approval from the Animal Care and Use Committee for Biosciences for the University of Alberta (Protocol number 682/12/11), the University of Calgary Life and Environmental Sciences Animal Care Committee (BI11R-10). Chickadees were captured under an Environment Canada Scientific permit (Permit number 09-MB-SC027), an Alberta Sustainable Resource Development (Fish and Wildlife Division) Collection and Research permits (Permit numbers 47908 and 47910, and a City of Edmonton Parks Permit.

### Playback Stimuli

To obtain black-capped chickadee mobbing calls, male black-capped chickadees that were not used in the experiment were placed in a small sound-attenuating chamber (inner dimensions 58×168×83 cm; Industrial Acoustics Corporation, Bronx, New York, USA) and left undisturbed for 24 hrs. The following day, between 0900 and 2000 chickadees were presented with a stuffed mount of either a perched northern saw-whet owl (length = 175 mm, wing length = 91mm), great horned owl (length = 645 mm, wing length = 349 mm), or a red-breasted nuthatch (length = 130 mm, wing length = 67 mm) in a randomized order with each mount presented twice for 3 mins and 1 h between each presentation. All recordings were made only while the mount was visible to the black-capped chickadee and were conducted between April 1^st^ 2008 and June 19^th^ 2008. Birds were recorded using an AKG C 1000S condenser microphone (frequency response: 50–20,000 Hz; AKG Acoustics, Vienna, Austria), and a solid-state recorder (Marantz PMD670, D&M Professional, Itasca, IL, USA). Vocalizations from black-capped chickadees that called in response to all three stuffed mounts were used to create the mobbing stimuli. Individual northern saw-whet owl, great horned owl, and red-breasted nuthatch calls were selected from Voices of North American Owls (Cornell Laboratory of Ornithology, Ithaca, New York, USA), Stokes Field Guide to Bird Songs: Western Region (Time Warner AudioBooks, New York, New York, USA), National Geographic Guide to Bird Sounds (Cornell Laboratory of Ornithology, Ithaca, New York, USA), Bird Songs of Canada's West Coast (Neville Recording, Salt Spring Island, British Columbia, Canada), and Alberta Birding by Ear (Barbara Beck, Edmonton, Alberta, Canada). All vocalizations were lowpass filtered at 10,000 Hz in Goldwave (Goldwave, St. John's, Newfoundland & Labrador, Canada) to remove background noise and normalized using SIGNAL version 5.0 sound analysis software (Engineering Design, Berkeley, California, USA).

We generated two stimulus sets for each vocalization type (e.g. two sets of northern-saw whet owl calls). Each stimulus set consisted of three vocalizations from three different individuals (i.e. set one, individual northern-saw whet owl a-b-c; set two individual northern-saw whet owl d-e-f) within a 15 s window followed by 45 s of silence. This one minute of playback was repeated 30 times resulting in a period of 30 minutes with stimulus playback for each stimulus category. Stimulus sets were constructed as in previous studies [19] to produce a stimulus presentation that was as natural as possible for the species selected. Thus, the duration of the stimuli varied but this variation did not correlate with expected results of the playback design (i.e., high threat calls were not longer). Within the 15 s window that playback calls occurred, the duration of the stimuli were: black-capped chickadee mobbing calls made to a northern saw-whet owl ∼7100 ms; the calls of a northern saw-whet owl ∼3100 ms; black-capped chickadee mobbing calls made to a great horned owl ∼3400 ms; the calls of a great-horned owl ∼8400 ms; the calls of a red-breasted nuthatch ∼7100 ms; and reversed playback of the black-capped chickadee mobbing call to a northern saw-whet owl ∼7100 ms (the identical calls used above were reversed).

### Playback

Individual birds were housed overnight in a chamber in a modified home cage which contained three perches at the level of the speaker and four water bottles and two food cups located evenly at either end of the cage. Playbacks were recorded using an AKG C 1000S condenser microphone (frequency response: 50–20,000 Hz; AKG Acoustics, Vienna, Austria), and a solid-state recorder (Marantz PMD670, D&M Professional, Itasca, IL, USA). We randomly selected one of six playback conditions to present to individual adult black-capped and mountain chickadees in sound attenuating chambers: 1) black-capped chickadee mobbing calls made to a northern saw-whet owl (high threat); 2) calls of a northern saw-whet owl (high threat); 3) black-capped chickadee mobbing calls made to a great horned owl (low threat); 4) calls of a great-horned owl (low threat); 5) calls of a red-breasted nuthatch (threat control); 6) reversed playback of the black-capped chickadee mobbing call to a northern saw-whet owl (methodological control). There were three adult black-capped and three adult mountain chickadees in each playback condition. Sample size was selected based on power to detect the interaction effect between black-capped and mountain chickadee and playback condition using R 2.12.2 [20]. There were four adult hand-reared black-capped chickadees in each of groups 1, 2, 5, and 6.

Stimuli were played back through a speaker (Realistic Minimus-7 Cat. no. 40-2034; input 8 OHMS, 40 W max; Radio Shack, Fort Worth, TX, USA) and amplifier (Cambridge Audio A300; London, UK) with an mp3 player (Creative ZEN; Singapore). The amplitude was measured at the level of the perches from the centre position of the cage and playback amplitude was set to approximately 74 db with a sound level meter (Radio Shack 33-2055; Radio Shack, Fort Worth, TX, USA).We conducted the experiment before fall equinox in late August and early September when both *chick-a-dee* calling and *fee-bee* song production is low [21]. The playback was conducted in one of six sound attenuating chambers (inner dimensions 58×168×83 cm; Industrial Acoustics Corporation, Bronx, New York, USA). Recording began at 1000 every day with 30 min of recording before playback with the lights illuminated, after which playback commenced and continued for 30 min. Following the playback period the lights were extinguished for 1 h. By playing back the calls in a sound chamber to one individual at a time we were able to control for other vocalizations and behaviours that would normally confound the auditory responses in natural settings.

### Histology

Following the playback method just described, each bird was given an overdose of 0.03 ml of 100 mg/ml ketamine and 20 mg/ml xylazine intramuscularly (1∶1) and then transcardially perfused with heparanized 0.1 M phosphate buffered saline (PBS) followed by 4% paraformaldehyde. Following perfusion, the brain was removed and placed in 4% paraformaldehyde for 24 h and then placed in 30% sucrose in PBS for approximately 24 h until saturated. The brains were then frozen in dry ice and stored at −80°C until immunocytochemistry (ICC) for ZENK protein was performed. For each bird, a cryostat was used to collect forty-eight 40 µm sagittal sections from each hemisphere starting from the midline and proceeding laterally. Sections were then placed into PBS. We processed brains in batches such that one of each treatment group was processed in each batch, but we randomly selected the individual bird to be included from each treatment group. Sections were washed in 0.1 M PBS, incubated in 0.5% H_2_O_2_ for 15 min, and washed again in 0.1 M PBS. Next, sections were incubated in 10% normal goat serum for 20 h, followed by incubation in the primary antibody (egr-1, Santa Cruz Biotechnology, catalogue # sc-189; Santa Cruz, CA, USA) at a concentration of 1∶5,000 in PBS containing Triton X-100 (PBS/T) for 24 h. Sections were then washed in PBS/T and incubated in biotinylated goat-anti-rabbit antibody for 1 h (1∶200 dilution in PBS/T). Next, sections were washed in PBS/T, incubated in avidin–biotin horseradish peroxidase (ABC Vectastain Elite Kit; Vector Labs; Burlington, ON, Canada) for 1 h and washed in 0.1 M PBS. Finally, the sections were visualized using 3,3′-diaminobenzidine tetrachloride (Sigma FastDAB, D4418; Oakville, ON, Canada), mounted on gelatin-coated microscope slides, dehydrated in ethanol and protected with cover slips affixed with Permount (Sigma-Aldrich; Oakville, ON, Canada).

### Analysis

ZENK immunoreactivity (ZENK-ir) was quantified for three auditory brain regions: the caudomedial mesopallium (CMM) and the ventral and dorsal parts of the caudal medial nidopallium (NCMv, NCMd; Figure 2.). The lateral ventricle defined the dorsal, ventral, and caudal borders of NCM, and field L defined the rostral border. ZENK-ir in CMM was quantified in the same sections used for NCM and was assessed in the most caudal area bounded by the lateral ventricle and the caudal-ventral boundary of the mesopallial lamina (LaM). For each chickadee, eight sections per hemisphere were measured for ZENK-ir. Quantification began with the first section in which mesopallium was contiguous with the rostral portion of the nidopallium to ensure that the orientation of the nidopallium was correct. This section, and the next seven sections moving laterally, was then mounted in the correct orientation. For each bird, 16 images (0.20 mm×0.15 mm) of each brain region, eight per hemisphere, were captured using a Leica microscope (DM 5500B; Wetzlar, Germany) with a 40× objective and a Retiga EX*i* camera (Qimaging, Surrey, British Columbia, Canada) using Openlab 5.1 (Perkin Elmer Inc., Waltham, Massachusetts, USA). Images were captured from locations used in previous studies [22]. For CMM, an image was captured from the most caudal part of the region. For NCM, a dorsal image was captured from the most dorso-caudal part of NCM and a ventral image was captured from the centre of the ventro-rostral region in an area of relatively high immunoreactivity. This sampling method, from which we counted the number of immunoreactive cells following a semi-automated protocol using ImageJ (NIH, v.1.36b; 2), captured images from the areas with the highest density of immunopositive cells within these auditory regions. This method has reliably found differences in previous studies [22]–[24].
